# Welfare Technologies and Ageing Bodies: Various Ways of Practising Autonomy

**DOI:** 10.1155/2018/3096405

**Published:** 2018-06-25

**Authors:** Anne Marie Dahler

**Affiliations:** Department of Public Health, Research Unit for Health Promotion, University of Southern Denmark, DK-6700 Esbjerg, Denmark

## Abstract

Contemporary policy strategies frame welfare technologies as a solution for welfare states facing the challenges of demographic change. Technologies are supposed to reduce or substitute the work of care workers and thereby reduce attrition among their ranks, reduce costs, and at the same make elderly people self-reliant and independent. In this paper, it is suggested that this way of framing how welfare technologies work with elderly people holds an instrumental view of technologies as well as of bodies and needs to be challenged. Drawing on an STS (Science Technology Studies) understanding of the constituting role of technology in people's lives, the guiding question in this study is how autonomy is practised in the lives of elderly people using welfare technologies. The study is based on interviews with eight elderly citizens in a Danish municipality who have been provided with a wash toilet and often also other technologies as part of their welfare service package. The study shows how autonomy is practised in various ways, how autonomy is practised in specific areas of life linked to the specific life story and body of the elderly citizen, how autonomy is situational as it is practised in specific situations during the day/week, and how autonomy is relational as it is practised in relation to specific persons and things and with specific persons and things. Implications of these findings are discussed in relation to the implementation of welfare technology as well as forms of governance appropriate for embodied elderly citizens and technologies.

## 1. Introduction

Welfare technology is a Scandinavian term for assistive technologies. The term is from 2007, and since then, there have been various attempts to define or delimit welfare technologies as distinct from “everyday technologies,” “medical technologies,” and so forth. The common understanding in those definitions is that welfare technologies are part of welfare services directed at citizens or supporting welfare professionals in providing services [1]. Welfare technology refers not only to specific kinds of technologies but also to a problem complex for which welfare technologies are expected to be a solution: an ageing population with more chronic diseases, fewer resources, and fewer hands to take care of their needs, often designated as “the elder burden” [2–5]. Welfare technologies are, alongside science and innovation, expected to be a means of solving this demographic challenge. The wash toilet, which is the technology focused on in this article, is explicitly mentioned in the Danish Strategy for Implementation and Dissemination of Digital Solutions and Welfare Technologies issued by the Danish government, the regions, and the Danish municipalities in 2013 [6]. This strategy states the following:

For citizens who need help using the toilet, a wash toilet can be an important tool to enhance self-reliance and dignity. At the same time, need for personal care is reduced, and the caretakers in the municipal home care will reduce their work load in drudgery positions. Automatic wash toilets are therefore relevant for citizens in their own homes and care centres. [6, p.13]

The wash toilet is a seat placed on top of an ordinary toilet. It has water jets for washing, air for drying, and a remote control with which the user can activate the washing and drying functions. Together, washing and drying take approximately 6-8 minutes.

In the municipality where the empirical work for this article was carried out, the local strategy on welfare technology is aligned with the national strategy and states that welfare technologies will provide citizens with the opportunity to live a self-reliant and independent daily life and reduce their dependency on public services and benefits [7]. The strategy states that the wash toilet is for people who need assistance with “lower hygiene” and that it provides for well-being and a feeling of freshness. It further states that, for employees, welfare technologies are associated with reduced physical strain. Such technologies are also expected to reduce costs for the municipal economy [7]. This article focuses on the relation between welfare technologies and the making of autonomous citizens.

The link between welfare technology and self-reliance, often expressed in terms of autonomy or independence, is a focus in various other studies, mainly in relation to mobility technologies [8–11] and often framed in terms of more or less dependence [9], that is, as a goal against which technologies are evaluated.

Independence is not always viewed uncritically as a goal for welfare technologies. Some studies point to ambivalences between independence and other values or goals, such as when the wheelchair enables the elderly citizen to go around on her own, while on the other hand she must surrender social interaction with her caregiver and becomes too tired to do other things during the day [12]. Long [13] frames independence as a policy goal that must be seen in the context of new international normative models of ageing, advocated by states, welfare workers, and others. The norm of independence, she holds, contributes to a public understanding of dependency as a burden, which means that one must incorporate technologies to remain a full adult person. Also, Struhkamp [14] points to independence as a policy construct, interpreted liberally, in which the autonomous individual exists as separate from social relations and is self-sufficient. In her study of patient autonomy, she relies on alternative interpretations of autonomy which emphasise relationality, interdependence, and vulnerability and show how patient autonomy unfolds in the activities of care [14].

Also, Gomez et al. [15] call for interpretations of autonomy that are not bounded in the dichotomy of dependence and independence. Based on a study of monitoring technologies, Gomez and colleagues show that an active engagement with these technologies sometimes enables autonomy but not always. They underline that it is an ethical challenge that technologies designed to promote autonomy do not always match the user or that use of the technology does not necessarily lead to autonomy [15].

With an increasing focus on the role of welfare technology as part of the solution to challenges of demographic changes, there is a need to investigate the role of specific welfare technologies in providing or supporting the autonomy of elderly people. This article attempts to contribute to the addressing of this need by attending to the wash toilet.

The guiding question in this study is how elderly people practise autonomy using welfare technology, with a specific focus on the wash toilet. To comprehend how elderly people practise autonomy with and by welfare technologies, it is argued that it is beneficial to discuss critically the idea of autonomy as self-reliance and the instrumental view of bodies and technologies that underlie policy strategies [16]. Drawing on an STS (Science Technology Studies) understanding of the constituting role of technology in people's lives, it is found that notions of autonomy vary—not only in theory and policy but also among elderly citizens. Furthermore, autonomy is relational as it is practised in relation to and with specific persons and things or technologies; autonomy is situational as it is practised in specific situations during the day and week, and autonomy is practised in specific areas of life linked to the specific life story and body of the elderly citizen. It is suggested that welfare technologies, qua their materiality and their constitution in specific sociopolitical practices, are actively engaged in changing these practices.

## 2. Materials and Methods

The question of how elderly people practise self-reliance and autonomy using welfare technology is addressed empirically by attending to when and how wash toilets are involved in practices of autonomy. The framework for collecting and analysing data is situational analysis (SA), a method of analysis inspired by grounded theory, but among others based on sociomaterial constructivism [17–19]. This approach foregrounds relations between heterogenous (human and nonhuman) actors and is thus suitable for studying relations of elderly people to other people and technology.

The study is designed as an interview-based investigation, based on eight semistructured interviews with elderly citizens who were provided with a wash toilet as an element of welfare service in a large Danish municipality. In Denmark, municipalities are the main providers of welfare technology, either by social service legislation or as part of a project. Municipality home-care unit managers mediated contact between project workers and the elderly. As interviews were conducted at the beginning of this project, all prospective participants able to participate were asked whether they wanted to take part in the study. Five of the elderly informants received the toilets as part of a project in which the toilets were distributed as a workplace device for employees under the Law on Work Environment. Three of the informants applied for a toilet under the Law on Social Service. Selection of informants was pragmatic in the sense that, at the time the empirical work was carried out, few people had received a toilet. Inclusion criteria were that the informants should be 70 years old or older and be able to take part in an interview. Elderly people suffering from dementia were thus excluded from the study.

The interviews took place in the homes of the participants and lasted between 1 and 3 hours. These meetings also included observations. Written informed consent was obtained from the participants for publication of this study. Interviews were carried out as semistructured interviews, inspired by Holstein and Gubrium's approach to interviewing, “Active Interviewing” [20]. In this approach to interviewing, the interviewer as well as the informants are understood as active creators of the interview situation and thus of the empirical material. Validating questions were posed during the interviews to validate the interviewer's understanding of the situation.

At the outset of the interview, the informant was asked about the functioning of the toilet and to show how it works. The interviewer (the author) also asked about other assistive devices/welfare technologies in their homes, the home-care service, their relatives, and their daily life (interview guide is found in Table 1).

The interviews were recorded, transcribed, and coded using a mix of thematic and in vivo codes. With inspiration from SA [18] messy maps, situational maps and relational maps were drafted for each informant. From these, illustrative case stories were drafted. The analytical questions posed to the material were as follows: When and how do elderly people practise autonomy and what is the role of wash toilets and/or other welfare technologies in these practices? After eight interviews, sufficient material was obtained to unfold the upcoming themes.

The research questions subsequently guided the analysis, with attention to when and how wash toilets are involved in practices of autonomy. Chunks of data and analysis of data have been presented to and discussed with fellow researchers from the “methodology group” at University College Lillebælt (UCL). It is a limitation of the study that only elderly users are included as informants. The empirical investigation of relational issues would have been strengthened by also including interviews with care professionals and with relatives.

## 3. Results and Discussion

The eight informants are between 70 and 84 years old, six of them more than 80 years old. Five are women, and three are men. They all live in the same municipality. Three women and two men received the toilet as part of a project in which the toilets were distributed as a workplace device for employees under the Law on Work Environment. These informants all received eldercare from the municipality (help with dressing, bathing, cooking, etc.). One man and two women applied for the toilet under the Law on Social Service. These three informants did not receive eldercare from the municipality. The analysis is built on interviews with all eight informants, but in the following section, case stories of two informants are presented to illustrate how matters of autonomy are braided into the specificities of their identities, bodies, and relations to people and things around them. Following these case stories, the results and discussion are organised into three sections. In the first section, “*less than ideally autonomous' elderly citizens*” notions of autonomy are discussed, as what appears to be autonomy varies among the informants. The second section,* ageing bodies as social flesh*, points to autonomy as relational, situational, and associated with embodied identities and lived lives. Although the use of wash toilets and other technologies is also discussed in these two sections, the third section,* the agency of (welfare) technologies*, addresses specifically the role of technologies in practising autonomy.

## 4. Henry

Henry was born in 1931 (interviewed November 2015). He lives alone in senior housing since his wife died six years ago. He has five adult daughters. They often take him out, he says. In the summer, they take him to the coast and eat eel, and once a week his youngest daughter takes him to the nearby shopping centre where they have breakfast in the cafeteria. He says that his health is just fine; it is only his legs that fail, so he does not walk well anymore. Three times a day, a home-care assistant comes to help him dress, shower (once a week), and do some cooking and housework. Not long ago, Henry received a wash toilet. He does not remember exactly when nor does he know why. However, he was going to have a new toilet, as the old one was too high, and “then, they came with this one.” Henry likes it because sometimes he experiences difficulties in wiping his behind. Henry shows me how the wash toilet works: “Perfect... you just need to wipe after with a cloth or toilet paper. The toilet takes most of it, and it also protects your underwear, when we reach the age that we have reached.” Henry is fond of the home-care assistants, whom he several times in the interview mentions as “the girls.” “If you are nice to them, they are nice to you”, he says. They come by to borrow his toilet because it is so nice; the seat is heated. He also likes to offer them a cup of coffee. During the interview, a male care worker passes by and uses his toilet. “There you can see; this is how we are related.” They come when you call, he says, and he tells about a situation where he had an accident [defecated in his trousers], while he was shopping with his daughter. Then he used his emergency call, and they were at his house and fixed the situation when he returned. During the interview, Henry pays attention mainly to his electric scooter, proudly showing it to me. He can drive around himself, without being dependent on anybody. He can go to the shopping centre himself; there is always somebody there to help him get goods off the shelves, both employees and other customers. Usually, he is there with one of his daughters who helps him. He prefers to use his scooter rather than take the bus for the elderly; “Then they [the bus driver and the other elderly passengers] don't need to be dependent on me.” He would not do without his scooter. He goes out with the scooter every day. He is an old sailor, he says, and he needs to go out in the “bush.” He tells stories from when he was sailing to and from South America and Africa. When the scooter is away for repair, he feels disabled. Henry once tried to sit in a manual wheelchair, but he hates wheelchairs, he says. He explained that his wife was linked to a wheelchair for many years. Then he said, “You will never get me in a wheelchair.”

## 5. Emily

Emily was born in 1934 (interviewed in autumn 2015). She lives in a rented house with a view of a small river. She has lived in the house since she was divorced nine years ago. When I came to her house, she was sitting on the floor, trying to take off her riding boots, and she asked me to help her. She had just arrived from a class in horseback riding. She then crawled towards her walker, stood up, and walked to the living room. Besides suffering from Parkinson's disease, she had a knee replacement a few months ago, due to rheumatoid arthritis. It was after the surgery that she acquired the wash toilet. “I am a nurse,” she said, and that is why she knew about the wash toilet. She describes the time after coming home from the hospital as horrible. She could no longer sense when she had to go to the toilet. She had to use diapers and needed to take showers and wash her behind several times a day, which took her “100 years,” as she had to take off trousers and stockings. She was in pain, and her medication for Parkinson's was complicated by painkilling medication. Emily felt she needed help, but the municipality sent her “unskilled employees” (home-care assistants). Had they just sent her home-care nurses, they would have understood her pain and her complications, she explained. She had been the leading home-care nurse in another municipality, and she was not satisfied with the help she had received. Also, they would not do the laundry for her, which she felt she needed after becoming incontinent. She applied for a wash toilet which she received two weeks before the interview. It makes a huge difference, she says. She feels much purer, and she does not smell like “old fish” any longer, she said. She also said that it prevents her from getting bladder infections. Now, she does not have home care from the municipality, and she would rather have her house stuffed with robots than have home-care assistants in her home. A cleaning assistant cleans her house every week. She shops for groceries on the Internet, and the guys who deliver the groceries are “so nice”. They even put it in her refrigerator and take the empty bottles and so forth with them. She has a Mac computer, an iPad, and an iPhone, and if there are any problems, Tom from the local Mac store comes to help her. She uses the computer for, among other things, searching for recipes and Skyping with friends. Emily cooks for herself, and once a week she has dinner with her two neighbours, a woman in her 30s and a man in his 40s. Age does not mean anything in their relations. However, age bothers her in other ways and situations. She said that one cannot do the same things anymore. “I get really angry if you are devalued because you are elderly and pushed aside and treated like... an elderly,” she said. Emily has two adult children who live with their families far away. She does not see them often as they have their own lives. She would like to see them more, but she also said that they should live their own lives, and she is happy that they are successful in what they are doing.

## 6. Less Than Ideally Autonomous Elderly Citizens

The two case stories above show that autonomy is practised in different ways and with regard to different activities in the lives of Henry and Emily. Foremost, they show that different notions of autonomy are at stake. For Emily, being able to take care of herself without help from home-care assistants is very important. For Henry, being able to get around on his electric scooter on his own makes him feel independent. In this section, various notions of autonomy will be discussed in relation to the empirical material.

Welfare technology is supposed to do something for the self-reliance of elderly people, and elderly people are, therefore, defined from the outset as “less than ideally autonomous” (expression borrowed from Agich [21]). Self-reliance is one of several similar terms used to denote autonomy [15] or independence [22] and can be conceived of as “the capacity to provide for one's own needs” [21]. In professional care work, autonomy—in terms of self-reliance—has been associated with the ability to do things without assistance, like cooking, cleaning, washing, making the bed, writing, and so forth. Some have criticised this understanding because it tends to victimise people with disabilities who define independence as an ability to be in control of and make decisions about one's life [22]. The distinction between executive autonomy (self-reliance) and decisional autonomy has led to new notions of care in which the voice and the will of the individual have been placed at the centre of care work and care policy [15].

However, Reindal [22] states that both notions of executive autonomy (self-reliance) and decisional autonomy are deficient, as they sustain a dichotomy between independence and dependence [22]; autonomy is attributed to the ability of the individual and thus located in the single, separate subject [22]. The individualistic view on the subject has been criticised by a wide range of scholars and positions defying liberal policy notions of autonomy, referring to individuals as existing separately from social relations and being self-sufficient [23, 24].

Self-reliance—understood as mentioned above as being able to do things without assistance—is explicitly mentioned as the goal and promise of welfare technology in public strategies [6, 25]. At the same time, the Danish professional conceptualisation of rehabilitation, which is worked out by professionals in the field of rehabilitation and which in principle has become a guideline for care work in all municipalities in Denmark, points to a broader range of values in care work. The goal of rehabilitation is that the citizen obtains an autonomous and meaningful life [26], and it is clarified that “autonomous” means that the individual will be able to control her life to the extent that she wishes, to the extent that is possible, given her resources and within the frame of relevant legislation [26]. Autonomy is framed in terms of personal preferences rather than self-reliance, and the definition of rehabilitation recognises interdependencies as the basis for living a meaningful life.

Turning to Henry, he does not measure up on the “self-reliance scale” as he needs help with dressing, washing, cleaning, cooking, and shopping; home-care assistants come to his home four times a day. His daughters, employees in shops, and other customers help him with shopping. He seems to be very content with the people around him, and he does not express any violation of his decisional autonomy, although he was not involved in the decision of having a wash toilet. Also, he seems to be in control of his life to the extent that is a practical possibility given his resources and the legal and organisational conditions framing the home-care service. What about Emily? Now that the first painful weeks after she was discharged from the hospital have passed, she is on her own. She has somebody come to clean her house; she orders grocery on the Internet, and when her computer fails, the nice guy from the Mac shop comes by her home. Also, when she goes horseback riding, she goes by taxi. In the sense that she does more housework than Henry does, she could be considered more self-reliant than Henry, but—on the other hand—she still needs assistance with daily chores, and she organises her daily life so she gets the assistance she needs.

The aim here, though, is not to compare the degree to which the interviewees are self-reliant. The point is that how self-reliance/autonomy/independence are conceptualised means something in terms of how people with disabilities and elderly people are constructed as less than autonomous. Furthermore, there is a clash between self-reliance as a policy goal and the aims of rehabilitation in care work. Birkmose [27] states that in rehabilitation practices there is a move from the professional notion of rehabilitation towards the goal of self-reliance in terms of being able to handle one's life without needing assistance from the welfare state, that is, home-care services. In this understanding, Henry will be constructed as low on the self-reliance scale of value, but Emily would perform very well. She organises and pays for her assistance herself, and she does not rely on home-care assistants in her everyday life, apart from the weeks after she was discharged from the hospital. Autonomy, then, is measured against the extent and costs of welfare services in one's life and home.

With attention to long-term care, Agich [21] questions the idea of autonomy as an abstract principle for long-term care. He proposes an ethical analysis of the nature of actual autonomy predicated on a concrete understanding of the everyday experience of autonomy and shifting attention to the actual manifestations of autonomy in everyday life [21]. In her study on patient autonomy, Struhkamp [14] also directs her attention to how autonomy is unfolding in the actual activities of care. Her interpretation of autonomy emphasises relationality, interdependence, and vulnerability, and in her study she shows how patient autonomy unfolds in specific activities of care [14]. Following this line of thought, the next section unfolds this relational aspect of autonomy by attending to how bodies and identities are from the outset related.

## 7. Ageing Bodies as “Social Flesh”

The case stories of Henry and Emily also point to autonomy as associated with their life stories and identities, that is, notions of who they are and who they were. The notion of autonomy (and thus self-reliance) informing most public policy is, according to Bacchi and Beasly [28], associated with an instrumental and singular notion of the body. They argue that the ways bodies are conceptualised matter to citizenship and find in their studies a demarcation line between those who seemingly control their bodies and those who do not, which also is a demarcation between full and lesser citizenship and between active and passive citizens.

How bodies are conceptualised in public policies have implications both in terms of government responsibilities associated with controlling the spaces in which citizen bodies operate and, thus, deciding what kind of services should be provided for these bodies and regarding how citizens think of themselves and their relationships with others. Bodies, then, are crucial to the understandings of autonomy and citizenship, and Bacchi and Beasley propose that citizenship and public policies should be informed by a fleshy, social body [28]. This fleshy, social body is central to the understanding of subjectivity in, for example, phenomenology, feminist studies, and postphenomenology. Subjectivities are embodied in the materiality of the body (the flesh), and bodies are embedded in relation to other bodies and the world (the social) [24, 29–31]. Subjectivity/identity is made possible precisely by the intercorporeal connections that all human beings sustain with each other [30].

This fleshy view on subjectivity defies a mere social constructivist approach to the body, in which ageing or age becomes a discursively produced construct. As Twigg [32] states, ageing is not an option, and in the same vein, old age is about nothing but the body:* “It swamps all other factors in determining matters like morale and well-being”* [32]. Ageing is also a material process, and the materiality of the body changes, so do the interconnections with other bodies and objects. The physical decline that accompanies ageing is often presented as a point of transition between a third age in which agency is emphasised and a dark fourth age of declining health and loss of agency and bodily self-control [33]. Receiving personal care can be understood as a key marker in this transition, as receiving help with getting washed, moving, eating, and excreting erodes the personhood and adult status of the subject [34]. The symbolism of this transition is, according to Twigg, particularly strong if a person suffers from incontinence: “To have an unbounded body in the context of modern expectations of the clearly bounded, individually defined body is to have one's autonomy and personhood questioned” [34]. With bodies that age differently and are embedded in various and varied relations and dependencies, autonomy is also practised differently in elderly people's lives. Henry can live in his house because the home-care assistants visit him four times a day. He does not seem to experience his relations with them as mere relations of dependency; he is nice to the caretakers, and they are nice to him.

The home-care assistants pass by and use his toilet or come for a cup of coffee; he is also offering something to the relation—kindness, coffee, and a toilet. When he spoke about his daughters, he said that they all became educated; he had given them something, and now they are also giving him something. In this sense, he does not seem to experience or practise these relations in terms of dependency-independency, and thus it does not seem important to him to practise self-reliance within these relations. However, when it comes to getting around, autonomy is important to him. As an old sailor, which is still a part of his identity, he is not to be pushed around in a wheelchair. Here, his experience of his wife in a wheelchair also interferes. He needs to go around on his own on his electric scooter, even if this implies that other people must go along (his daughter, other customers) and assist him in picking groceries from the shelves. So, Henry is practising self-reliance in relation to the one arena that is important to his identity, the arena of “getting around”.

Emily is practising autonomy by going on with everyday practices to the extent that she can. She does not want to be dependent on home-care assistants, and her identity as a nurse and a former leader of home care in a municipality frames how she views them, as unskilled labour. So, in a sense, Emily is practising self-reliance by keeping home-care assistants from the door. She is dependent on help from others in terms of shopping, cleaning, and transportation, which she organises herself, and she is networking with her neighbours in a setting in which she feels that age is not an issue. Ageing for her is a burden in terms of not being able to do what she was used to, and she suffers from the devaluation she experiences as elderly. In this sense, she is practising self-reliance by living as “normally” (as in not being old) as possible and not like an elderly person in need of home-care assistance.

Another elderly woman, Ellen, who is also in her eighties, is living on her own in a housing complex for elderly persons. She suffers from many things, is very obese and can hardly move, and has, over the last eight to nine years, also become blind. She seldom leaves her apartment. Home-care assistants come four times a day and help her with bathing, dressing, and so forth, except for cooking. She does not want the food from the municipality, which she describes as terrible. One of her daughters brings her home-cooked frozen food a couple of times a week, which the home-care assistant can heat in the microwave. She is practising autonomy then by deciding what to eat. Moreover, her daughter is helping her practise autonomy. Ellen has been on local TV lately speaking in opposition to budget cuts on eldercare in the municipality. She had been lying in her bed until midmorning with a wet diaper; she suffered from eczema in the crotch and wanted public attention on how the elderly were treated. She said that other elderly were afraid to speak out in public, fearing consequences such as reduction of care. Speaking out in public about the care she receives is also a way of practising autonomy. Ellen was provided with a wash toilet to get rid of the eczema. After she had the toilet, she was not supposed to have help with “lower hygiene,” as it is called in the municipal system. However, she has “tamed” the home-care assistants to wash her in the crotch every morning and to apply ointment. With the toilet alone, she cannot get rid of the eczema. She talks about her demands to the home-care assistants as a matter of taming them or telling them what to do. So, she is practising autonomy towards and together with the assistants on whose care she depends, and here autonomy is opposed to self-reliance.

Inga received her wash toilet after she broke an arm. The other arm did not work very well, so she could not wipe her behind. She lives alone in the house that she and her late husband built many years ago. She did not have any home care until she broke her arm. She had to call home-care service while she was sitting on the toilet to come and wipe her, and it sometimes took them a half an hour to arrive. The waiting time annoyed her, as did the fact that they sometimes sent male home-care assistants with non-Danish ethnicity. She did not mind Danish female home-care assistants wiping her, but she did not want to be dependent on these non-Danish males. So, she was very happy with her wash toilet. When she was going out, for example, to concerts, her friends would help wipe her behind when she had to go to the toilet. She did not mind that; that is a part of becoming older. Therefore, being or becoming dependent on others seems to be a problem for Inga mainly when the helper is a non-Danish-ethnicity male who has to wipe her behind and not when these others are her friends.

Then there is Harry. Harry lives with his wife. He has Parkinson's disease, and one of his arms does not work. His wife applied for the wash toilet because Harry depended on her help to wipe his behind. It was also his wife who figured out how the toilet worked, and she was standing beside him the first few times he used the toilet. Both Harry and his wife are thus users of the toilet, and the toilet provides both with some independence. Harry's wife does not have to be there when Harry goes to the toilet, and Harry does not need the help of his wife at that time.

These examples show how experiences and practices of autonomy are embodied in (very different) fleshy identities of elderly people and embedded in various constellations of dependencies and independencies. Autonomy is relational, as it is practised in relation to or together with specific persons (when Ellen's daughter is supplying her with frozen meals, so she does not have to be dependent on meals from the municipality; when Inga is going to a concert and her friend will help her wipe her behind; and when Harry is enabled to wipe his behind without help from his wife). Autonomy is situational as it is practised in specific situations during the day/week (when Henry is going out, when Ellen is taming her home-care assistants or talking on local TV). Also, autonomy is practised in specific areas of life linked to the specific life story and body of the elderly person (Henry the sailor and his scooter, Emily the nurse and the home-care assistants). Moreover, autonomy is also practised with and without help from welfare technologies, as is mentioned several times, for example, when Henry is practising autonomy with help from the electric scooter, and Emily and Inga are practising autonomy in relation to home-care assistants with the aid of the wash toilet.

## 8. The Agency of (Welfare) Technologies

This section addresses the role of welfare technology in enabling autonomy. The notion of technology is unfolded by attending technologies as active participants in everyday practices. Although conceptualised in various ways, specific attention is given in Science and Technology Studies to how technologies and other materialities coconstitute our lives and worlds. The agency of technologies is often referred to in terms of Akrich's [35] notion of a script. It infers that technologies may be analysed like a play in which characters are defined and roles and relations between actors (human and nonhuman) are set [35]. Various studies in the field of care technologies have used the notion of a script, often with a focus on the design of technologies, but also in analysing what values technologies embody for their users by attending to the practices in which they are used. Pols and Moser [36], for example, use “script” as a core concept in their study on affective relations between the user and technology when they investigate what kind of social and affective relations are enacted in use practices involving social robots [36]. The scripts of artefacts suggest specific actions and discourage others; “in the mediation of action, one could say that specific actions are ‘invited' while others are ‘inhibited'” [37]. In Emily's case, the wash toilet enables her to practise autonomy as it invites her to do the washing of her behind without the help of a home-care assistant. It enables her to maintain her role as a private and full person—a (former) nurse and leader—and enables her to practise autonomy understood as self-reliance, as she is almost independent of welfare services. In this sense, the wash toilet is inscribed with a specific relationship between Emily as a full citizen and the welfare state.

This does not mean that wash toilets always enable autonomy or self-reliance. Technologies are acting and being acted upon within particular practices and are, therefore, interacting with actors who have their own notions about the parts they and other actors have to play [36]. Technologies do something with their network or practise, and other actors do something in return. The script of the technology then does not reside in the technology per se, but in its relations and interactions within a specific set of practices [38]. Technologies interact with fleshy, material, related bodies, and identities. For Ellen, the elderly woman with eczema, the wash toilet has helped reduce the eczema, and now the home-care assistant washes her behind only once a day. In that sense, one could say she has become a little more self-reliant (a little less in need of welfare services), but her actual act of autonomy has been to insist on still being washed by a home-care assistant every morning. Henry likes his wash toilet, and he likes that the home-care assistants use his toilet. They pass by his home to use his toilet. So, the toilet enables a kind of relation between Henry and the home-care assistants other than a clear-cut professional patient-citizen relation. However, in Henry's life, autonomy seems to be more related to going around on his electric scooter than to bodily care practices. The electric scooter enables him to hold on to his identity as independent/sailor/mobile and to go around and do things on his own—most often with his daughter. In the words of postphenomenology, it is the human-technology associations in these cases that are practising autonomy, not Ellen and Henry, but the association of Ellen and the wash toilet and the association of Henry, the electric scooter, and Henry's daughter. So, not all welfare technologies are associated with matters of autonomy, and matters of autonomy are not unambiguously framed positively. For Henry, using a wheelchair would be the end of his life as Henry.

For Anna, associating with the welfare technologies in her home is also not unambiguously about practising autonomy. She has had home-care assistants coming to her home for 30 years, as she has lived with rheumatoid arthritis from a young age, and besides the wash toilet, she has a walker, a wheelchair, a care-bed, and some other devices. She is used to receiving home care. However, it bothers her that her brother and sister-in-law frame her use of technologies as a dependency on the welfare state. According to Anna, they would never themselves receive technologies or other services from the municipality. She says (with sorrow) that in their view her life equals living in a poorhouse. The themes and findings of the study are summarised in Table 2.

## 9. Conclusions

The point of departure in this paper is the public policy idea of the ability of welfare technology to make autonomous or self-reliant elderly citizens. Pointing to different meanings of autonomy and self-reliance, to the ageing body as a fleshy social body, and to the agency of technologies, the paper has shed light on how elderly people practise autonomy and on the role of the wash toilet in these practices.

The study shows how experiences and practices of autonomy are embodied in (very different) fleshy identities of elderly people and embedded in various constellations of dependencies and independencies. Often, autonomy and self-reliance go hand in hand but not always. Autonomy is practised in specific areas of life linked to the specific life story/body/identity of the elderly person; autonomy is situational as it is practised in specific situations every day, and autonomy is a relational process as practised in relation to or together with specific persons. Autonomy is sometimes practised in association with welfare technologies and sometimes not. Welfare technologies actively engage in various aspects of the lives of the elderly and their relations to other people and the world around them, sometimes in the battlefield of autonomy and sometimes not. Autonomy is enabled by the materiality of the technology but produced as the effect of networks of heterogeneous actors, including this materiality, elderly identities/bodies, relatives, caregivers, and other actors who or which have not been the focus of this study: professional ideologies, visitation practices, organisation of home care, legislation, and all other arrangements that make up the lives/days of elderly citizens. Also, qua their materiality and the practices in which they are used, they can enact specific normativities and normative ideas of how elderly people should live their lives.

The study calls for attention to the notions of autonomy involved in policies and strategies regarding welfare technologies and how they construct elderly people as fuller or lesser citizens based on their eventual needs for welfare services and care. It calls for a more humble and nuanced use of terms such as autonomy and self-reliance in those policies as it reveals that autonomy is practised in various ways and areas of life in elderly people's everyday practices, and it sometimes involves the work and services from caregivers. In implementation processes, attention should be paid to the elderly as a fleshy social body, how technologies will associate with this body, and the various arrangements in which this specific body is situated. In this sense, the study challenges the idea of large-scale implementation of specific welfare technologies as it ignores the specificities of bodies, identities, and technologies which have been demonstrated vividly in this study. The study also calls for public policies that capture the full complexity of social flesh as well as the agency of welfare technologies. The Danish notion of rehabilitation that recognises interdependencies as the foundation for living an autonomous and meaningful life could be a starting point if autonomy is not reduced to self-reliance in terms of being able to get along without welfare services. More research is needed regarding how autonomy is practised within the framework of rehabilitation work. Further inquiry may start from the interactions of welfare technologies, elderly people, relatives, and care professionals, among others, and focus on how and when autonomy and self-reliance go together and how and when they oppose one another.

## Figures and Tables

**Table 1:** Interview guide.

**Citizen**	**Notes**	**Reflections**
Date/time		

Who is there		

Where		

Case No.		

Introduction, presentation, aim		

Presentation of informant		
Tell me about your day?		
When and why did you receive the wash toilet?		

Do you know why wash toilets have been implemented in the municipality?		
How were you informed?		

How were you prepared?		
(i) By whom?		
(ii) In which ways?		

What was your reaction towards the idea of having a wash toilet?		

Tell me/show me how it works?		

Tell me about actual situations where you have been happy with/not happy with the wash toilet?		

What are your expectations from the wash toilet? What will it do for you?		

Other technologies in your home?		

**Table 2:** Welfare technology, autonomy, and ageing bodies—themes and findings.

**Themes – aspects of autonomy**	**Findings**
Notions of autonomy vary	Elderly people hold different ideas of autonomy (as do theory and policy). Autonomy is not always an important issue for elderly people

Autonomy as relational	Autonomy can be practised in association with other people and technologies, and by insisting on help from home-care personnel (clash between self-reliance and self-determination)

Autonomy as situational	Autonomy is practised in specific situations. Autonomy is not always important in toileting situations

Autonomy as linked to life story and body of elderly	Whether and when autonomy is important seem to be associated with the specific embodied identity and life story of the elderly person

Welfare technology can, but does not always play a part in practices of autonomy	Technologies can in specific situations support the autonomy of specific elderly persons, with specific embodied identities

## Data Availability

The qualitative data set is not publicly available, as the project is registered with the Danish Data Protection Agency and committed to keeping the informants anonymous.
